# *Parasaturnius maurepasi* n. gen. et n. comb. (Digena: Bunocotylidae) from the stomach of the silver mullet, *Mugil curema* (Perciformes: Mugilidae) in coastal lagoons of northern Yucatán, Mexico

**DOI:** 10.1007/s11230-023-10142-z

**Published:** 2024-01-22

**Authors:** Leopoldo Andrade-Gómez, Gerardo Pérez-Ponce de León

**Affiliations:** Departamento de Sistemas y Procesos Naturales, Escuela Nacional de Estudios Superiores Unidad Mérida, Km 4.5 Carretera Mérida-Tetiz, C.P. 97357 Ucú, Yucatán Mexico

## Abstract

Bunocotylid trematodes represent a group of 149 species with a rather complex taxonomic history. The current concept of the subfamily only includes three genera, *Bunocotyle*, *Saturnius*, and *Robinia*. Specimens of a bunocotylid were collected from the silver mullet, *M. curema*, from a coastal lagoon of Yucatán and identified as belonging to *Saturnius*. Further detailed morphological study revealed they corresponded to *S. maurepasi*, a species previously reported from the stripped mullet, *Mugil cephalus* in Mississippi, USA. Specimens were sequenced for the LSU of nuclear ribosomal RNA gene (28S) to test their phylogenetic position. We discovered that they do not belong in *Saturnius* since they nest as an independent lineage which is the sister taxa of a clade formed by *Robinia*, and *Saturnius* + *Bunocotyle*; additionally, the new genus exhibits high genetic divergence (10-12%) with respect to species allocated in the other bunocotylid genera. The species *S. maurepasi* was then transferred to the new genus as *Parasaturnius maurepasi* **n. gen., n. comb.** that was created to accommodate it, and was redescribed based on newly sampled specimens.

## Introduction

Members of Bunocotylidae Dollfus, 1950 are a relatively diverse group of trematodes with approximately 149 species infecting the gastrointestinal tract (mainly the stomach) of marine and estuarine fishes around the world (Atopkin et al., 2020; WoRMS, 2023). The taxonomic history and classification of the family has been rather controversial (Pankov et al., 2006; Sokolov et al., 2019; Louvard et al., 2022; Martin et al., 2023). Using a molecular phylogenetic analysis based on 28S rDNA, Atopkin et al. (2020) showed that Bunocotylidae was constituted by four subfamilies, namely Bunocotylinae Dollfus, 1950 with three genera, Hysterolecithinae Yamaguti, 1958 with four, Opisthadeninae Yamaguti, 1970 with five, and Quadrifoliovariinae Yamaguti, 1955 with three genera (see Worms, 2023). Nevertheless, in recent molecular phylogenetic analyses, these subfamilies of Bunocotylidae were yielded as paraphyletic (Louvard et al., 2022; Faltýnkova et al., 2022). In a recently published study, Martin et al. (2023) also suggested that Bunocotylidae was paraphyletic relative to the Hemiuridae *sensu stricto* and the Lecithasteridae *sensu stricto*. Based on the latter arguments, the current concept of Bunocotylinae contains only 14 species classified in three genera, *Bunocotyle* Odhner, 1928, with four species; *Saturnius* Manter, 1969, with nine; and *Robinia* Pankov, Webster, Blasco-Costa, Gibson, Littlewood, Balbuena & Kostadinova, 2006, with only one species (Pankov et al., 2006; Blasco-Costa et al., 2008; Louvard et al., 2022; Faltýnkova et al., 2022).

Members of this subfamily are mostly parasites of the stomach of mullets of the genus *Mugil* (L.) since 11 of the 14 species have been described in that fish group (Overstreet, 1977; Blasco-Costa et al., 2006; 2008; Marzoug et al., 2014). Only three species of bunocotylids have been described in the Americas, i.e., *Bunocotyle sudatlantica* Parukhin, 1976 from an unidentified fish belonging to Chaetodontidae Rafinesque, 1815 in Brazil; *Saturnius maurepasi* Overstreet, 1977 from the stripped mullet *Mugil cephalus* (L.) in Mississippi, USA; and *Saturnius belizensis* Fischtal, 1977 from the silver mullet *Mugil curema* Valenciennes in Belize (Kohn et al., 2007; Blasco-Costa et al., 2008; WoRMS, 2023). Overall, only four of the 14 species have been sequenced for some molecular marker, including *Saturnius gibsoni* Marzoug, Rima, Boutiba, Georgieva, Kostadinova and Pérez-del-Olmo, 2014; *S. minutus* Blasco-Costa, Pankov, Gibson, Balbuena, Raga, Sarabeev and Kostadinova, 2006; *Bunocotyle progenetica* (Markowski, 1936); and *Robinia aurata* Pankov, Webster, Blasco-Costa, Gibson, Littlewood, Balbuena and Kostadinova, 2006 (Pankov et al., 2006; Marzoug et al., 2014).

During a survey on the parasites of marine and estuarine fishes of the Yucatán Peninsula, specimens of the silver mullet, *Mugil curema* (Valenciennes) were collected in La Carbonera coastal lagoon, in Yucatán. Specimens of a bunocotylid trematode were obtained from the stomachs of their hosts. After comparing morphologically with the original descriptions of members of the family, we identified our specimens as *Saturnius maurepasi*, with a slight morphological variation. However, while conducting a molecular phylogenetic analysis of the large subunit of the ribosomal gene (28S rDNA), we unexpectedly discovered that our specimens formed a separate monophyletic clade from *Saturnius* spp., suggesting that they represented an undescribed genus. Here, we present the diagnosis of the genus *Parasaturnius* **n. gen.** to include *S. maurepasi* as a new combination, using morphological and molecular evidence, and we discuss the interrelationships within Bunocotylidae.

## Materials and methods

### Host collection and morphological analysis

A total of 10 individuals of *M. curema* (8–10 cm) were collected in May 2022 in La Carbonera coastal lagoon, Yucatán State, Mexico (21° 08′ 1.5′′ N, 90° 07′ 55.9′′ W) using cast nets; silver mullets were kept alive and examined for helminths a few hours after capture. Individual fish were euthanized by spinal severance (pithing) following the procedures accepted by the American Veterinary Medical Association (AVMA, 2020), dissected, and immediately examined under a stereomicroscope. Bunocotylids were recovered from the stomach lining of seven of the 10 host examined, fixed in hot distilled water, and preserved in 100% ethanol for morphological and molecular analyses.

Some unflatten specimens were post-fixed in hot formalin to harden the tegument. Specimens were dehydrated through graded alcohol series, stained with Mayer’s paracarmine (Merck, Darmstadt, Germany), cleared with methyl salicylate, and mounted on microscope slides with Canada balsam. Mounted specimens were examined under a bright field Nikon DS-Ri1 microscope. Measurements were taken using Nikon NIS Elements microscope software (Nikon) and are given in micrometres (μm). Drawings were made with Adobe Illustrator 25.4.1 (Adobe, Inc). Vouchers were deposited in the Colección Nacional de Helmintos (CNHE), Instituto de Biología, Universidad Nacional Autónoma de México, Mexico City.

For scanning electron microscopy (SEM), specimens were dehydrated in a graded ethanol series, critical point dried, and sputter coated with gold. Then, specimens were examined with a Hitachi Stereoscan Model SU1510 scanning electron microscope at 15 kV at the Laboratorio de Microscopia y Fotografía de la Biodiversidad, Instituto de Biología, Universidad Nacional Autónoma de México.

### Molecular analysis

Two bunocotylids were placed individually overnight in tubes with a digestion solution for DNA extraction at 56°C. The digestion solution contained 10 mM Tris-HCl (pH 7.6), 20 mM NaCl, 100 mM Na_2_ EDTA (pH 8.0), 1 % sarkosyl, and 0.1 mg/ml proteinase K. DNA was extracted using DNAzol reagent (Molecular Research Center, Cincinnati, Ohio). The domains D1–D3 of the large subunit of nuclear ribosomal RNA gene (28S) were amplified via PCR using the primers: 391 5′– AGCGGAGGAAAAGAAACTAA–3′, and 536: 5′-CAGCTATCCTGAGG GAAAC-3′ (García-Varela and Nadler, 2005). The amplification and sequencing protocols followed those used in Andrade-Gómez et al. (2023). Sequences were assembled and edited using Geneious v7 (Kearse et al., 2012) and deposited in the GenBank database.

The two newly obtained sequences were aligned with data from other members of Hemiuroidea downloaded from the GenBank dataset, plus one species from Azygiidae used as outgroup for rooting the tree (see Table 1). The final alignment consisted of 35 sequences with 1,326 nucleotides*.* Alignments were built using the software Clustal W (Thompson et al., 1997) with default parameters and adjusted manually with the Mesquite software (Maddison & Maddison, 2011).

**Table 1:** Sequences of 28S from GenBank used for phylogenetic analysis in the present study

Family	Species	GenBank accesion	Reference
Out group			
AZYGIIDAE	*Otodistomum cestoides*	AY222187	Olson et al. (2003)
Super Family HEMIUROIDEA			
GONOCERCIDAE	*Gonocerca phycidis*	KY197009	Sokolov et al. (2018)
	*Hemiperina manteri*	AY222196	Olson et al. (2003)
DEROGENIDAE	*Thometrema lotzi*	KC985236	Calhoun et al. (2013)
	*Derogenes varicus*	AY222189	Olson et al. (2003)
ISOPARORCHIIDAE	*Isoparorchis eurytremus*	MH628315	Sokolov et al. (2019)
LECITHASTERIDAE	*Lecithophyllum botryophoron*	MH628301	Olson et al. (2003)
	*Aponurus laguncula*	KU527430	Claxton et al. (2017)
	*Lecithaster gibbosus*	AY222199	Olson et al. (2003)
	*Lecithaster mugilis*	LN865016	Besprozvannykh et al. (2017)
Family unknown (Atopkin et al. 2020)	*Merlucciotrema praeclarum*	AY222204	Olson et al. (2003)
HEMIURIDAE	*Hemiurus luehei*	MH628316	Sokolov et al. (2019)
	*Lecithochirium microstomum*	KC985235	Calhoun et al. (2013)
	*Pulmovermis cyanovitellosus*	MH628314	Sokolov et al. (2019)
	*Plerurus digitatus*	AY222201	Olson et al. (2003)
	*Dinosoma synaphobranchi*	MH628302	Sokolov et al. (2019)
	*Brachyphallus crenatus*	MH628299	Sokolov et al. (2019)
	*Myosaccium ecaude*	OP918123	Pantoja & Kudlai (2022)
	*Parahemiurus merus*	OP918125	Pantoja & Kudlai (2022)
	*Aphanurus mugilis*	LT607807	Atopkin et al. (2017)
	*Lecithocladium excisum*	AY222203	Olson et al. (2003)
	*Ectenurus virgula*	OP918126	Pantoja & Kudlai (2022)
	*Dinurus longisinus*	AY222202	Olson et al. (2003)
BUNOCOTYLIDAE			
Bunocotylinae	*Robinia aurata*	DQ354367	Pankov et al. (2006)
	*Bunocotyle progenetica*	DQ354365	Pankov et al. (2006)
	*Saturnius minutus*	DQ354366	Pankov et al. (2006)
	*Saturnius gibsoni*	KJ010542	Marzoug et al. (2014)
Hysterolecithinae	*Machidatrema chilostoma*	AY222197	Olson et al. (2003)
	*Hysterolecithoides epinepheli*	MH628310	Sokolov et al. (2019)
Quadrifoliovariinae	*Bilacinia australis*	AY897568	Chambers & Cribb (2006)
	*Quadrifoliovarium simplex*	AY897564	Chambers & Cribb (2006)
Opisthadeninae	*Opisthadena dimidia*	AY222198	Olson et al. (2003)
	*Genolinea anura*	MH628308	Sokolov et al. (2019)

Phylogenetic analyses were performed using Maximum Likelihood (ML) and Bayesian Inference (BI) methods. ML was carried out with RAxML version 7.0.4 (Silvestro & Michalak, 2011) and Bayesian Inference analyses were run with MrBayes version 3.2.7 (Ronquist et al., 2012) using the online interface CIPRES (Cyberinfrastructure for Phylogenetic Research) Science Gateway v3.3 (Miller et al., 2010). The best model was estimated with the Akaike information criterion (AIC) using the jModel Test version 0.1.1 program (Posada, 2008), which predicted the best model for the 28S dataset to be GTR + I + G. Nodal ML support was achieved through 1000 bootstrap replicates. The BI analyses included Markov Chain Monte Carlo (MCMC) searches of two simultaneous runs for 10 million generations, with sampling every 1000 generations, a heating parameter value of 0.2, and the first 25% of the sampled trees were discarded. Trees were drawn using FigTree program v.1.4.4 (Rambaut, 2010). Uncorrected P distances were obtained in MEGA version 6 (Tamura et al., 2011).

## Results

**Class Trematoda Rudolphi, 1808**

**Subclass Digenea Carus, 1863**

**Order Plagiorchiida La Rue, 1957**

**Suborder Hemiurata Skrjabin & Guschanskaja, 1954**

**Superfamily Hemiuoroidea Looss, 1899**

**Family Bunocotylidae Dollfus, 1950**

***Parasaturnius*** **n. gen.**

**Type-species:** *Saturnius maurepasi* Overstreet, 1977.

**Diagnosis.** Body elongate, cylindrical, with maximum width at ventral sucker flange level. Tegument unarmed, with fine transverse striations. Two circular muscular flanges present around body. Anteriormost flange surrounds oral sucker. Second flange at posterior end of ventral sucker, forms 2 lateral subconical protuberances with concentric muscles. Body with 7 pseudosegments separated by 6 transverse fibrous septa. Oral sucker muscular, subterminal bearing small papillae. Large single cells present in segments.

Ventral sucker strongly muscular, sub-spherical, anterior to mid-body. Prepharynx absent; pharynx subspherical. Oesophagus short. ‘Drüsenmagen’ present. Caeca with constrictions at septa levels. Testes 2, smooth, in tandem, located at hindbody. Seminal vesicle large, saccular antero-dorsal to ventral sucker, larger than sinus-sac. Sinus-sac small elongate-oval, contains short muscular hermaphroditic duct. Pars prostatica vesicular, small, difficult to distinguish, enters sinus-sac at its base. Genital pore at level of anterior septum. Ovary transverse, oval, in anterior end of last pseudosegment, ventral to caeca, smooth to irregular, contiguous with vitellarium. Mehlis’ gland and uterine seminal receptacle present. Laurer’s canal not observed. Vitellarium compact, smooth to irregular, elongate-oval, larger than ovary. Uterus thin-walled, extends posterior to vitellarium. Metraterm short. Eggs numerous. Excretory pore wide, terminal or subterminal; vesicle Y-shaped.

**Etymology.** The genus *Parasaturnius* **n. gen.** refers to its resemblance with *Saturnius* Manter, 1969, and uses the Greek prefix *para* (meaning resemble).

### Remarks

*Parasaturnius* **n. gen.** can be differentiated from *Robinia* and *Bunocotyle* by the presence of pseudosegments (transverse fibrous septa) in the fore- and hind-body. Furthermore, the genus *Robinia* possesses fine striations, symmetrical testes, 11-15 muscular lobes on the oral sucker, and vestigial ecsome. The new genus differs further from *Bunocotyle* by the lack of cyclocoel. *Parasaturnius* **n. gen.** is morphologically very similar to *Saturnius* since both genera present pseudosegments. Nonetheless, the new genus can be distinguished by the presence of four pairs of small papillae surrounding the aperture of the oral sucker.

**Figure 1.**
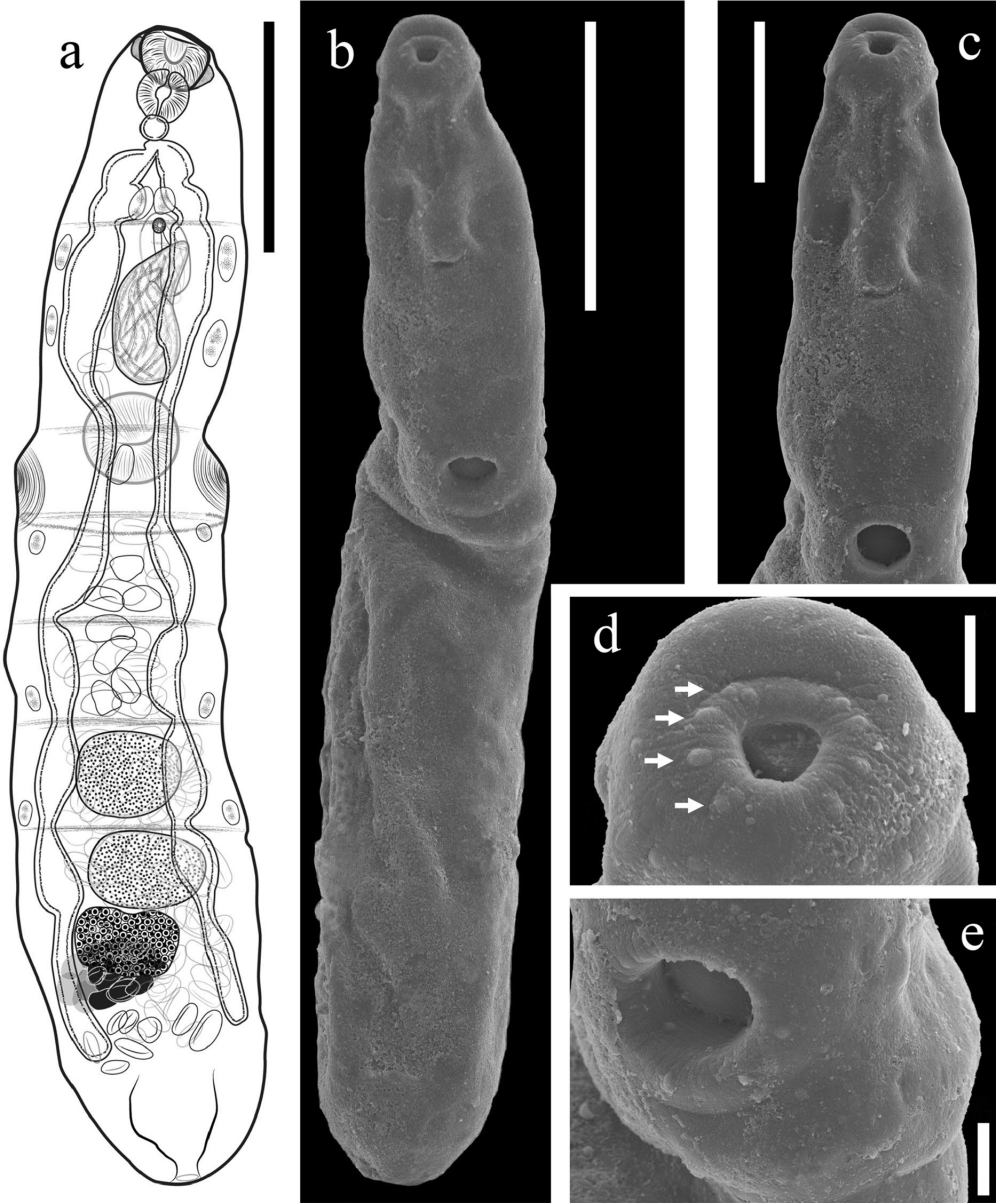
*Parasaturnius maurepasi* (Overstreet, 1977) **n. gen. n. comb.** from *Mugil curema* (A) whole worm voucher (Dorsal view); Scanning electron micrographs of voucher (B) Whole worm; (C) Forebody (D) Oral sucker, Arrows indicates papillae; (E) Ventral sucker. Scale bars (μm) = (A, B) 100; (C) 50; (D,E) 10.

***Parasaturnius maurepasi* (**Overstreet, 1977**) n. gen. n. comb.** (Fig. 1)

Syn. *Saturnius maurepasi* Overstreet, 1977

**Records**: 1. Overstreet (1977); 2. Romero & Galeano (1981); 3. Fernandes & Goulart (1992); 4. Knoff et al. (1997); 5. Present study.

**Type host**: *Mugil cephalus* L. (1, 2)

**Other hosts***: Mugil liza* Valenciennes, parati mullet (3, 4); *Mugil curema* Valenciennes, silver mullet (5)

**Type locality**: Ocean Springs, Mississippi (1)

**Other localities**: off Santa Marta, Colombia (2); Brazilian coast of SW Atlantic (3, 4); La Carbonera, Yucatán State, Mexico (5)

**Site of infection**: Stomach lining

**Prevalence**: 70% (5)

**Intensity**: 1-6 (5)

**Specimens studied**: 10 voucher specimens (CNHE 12843)

**Representative DNA sequences**: OR831227– OR831228 (28S)

R**e**description (Based on 10 mature individuals. Measurements in Table 2)

**Table 2:** Comparative metrical data for *Parasaturnius maurepasi* from America in different hosts plus *S. belizensis*

	*Parasaturnius maurepasi* **n. gen. n. comb.**	*P.* (*Saturnius*)* maurepasi*	*P.* (*Saturnius*)* maurepasi*	*P.* (*Saturnius*)* maurepasi*	*S. belizensis*
Reference	This study	Overstreet, 1977 (Original description)	Blasco-Costa et al. 2008	Fernandes and Goulart, 1992	Fischtal, 1977; Blasco-Costa et al. 2008
Locality	La Carbonera, Yucatán State, Mexico	Mississippi sound, USA	Mississippi sound, USA	Rio de Janeiro, Brazil	Belize city shore, Belize
Host	*Mugil curema*	*Mugil cephalus*	*Mugil cephalus*	*Mugil liza*	*Mugil curema*
n	10	37	5 paratypes	6	2
BL	430−616 (510)	497−1085	782−1098	630−830	415−694
NPSG	7	7*	7	6*	5
BWatVSF	63−103 (80)	77−139	120−135	120−150	65−84
BWatVS	31−78 (59)	106*	101−122	102*	64
FO	126−200 (157)	313*	237−316	205−261	155−189
LPL	131−190 (161)	173−373	173−376	291*	120−195
LPW	75−141 (92)	153*	150−165	152*	103
OSL	20−35 (28)	29−42	36−41	32−35	28−29
OSW	27−44 (34)	33−48	38−48	38−47	30−31
VSL	23−49 (39)	46−75	62−69	40−68	40−45
VSW	37−48 (43)	49−77	67−77	56−68	41−44
FatVSL	21−38 (27)	33*	30−51	35*	35
FatVSW	9−20 (14)	13*	10−16	13*	13
PHL	17−28 (22)	22−32	28−34	23−30	18−22
PHW	17−27 (21)	19−33	26−32	23−33	18−24
OEL	9−20 (13)	26*	−	11	25−30
SSL	28−47 (35)	47*	28−39	26−33	22−44
SSW	20−38 (27)	21*	11−24	16−23	15−17
SVL	47−75 (56)	97*	82−146	84*	65
SVW	30−38 (34)	23*	32−50	44*	39
PPL	12−21 (14)	15	15	−	21*
PPW	11−20 (14)	11	11	−	34*
ATL	37−63 (49)	28−55	43−64	28−35	35−48
ATW	40−60 (51)	30−62	50−84	54−66	38−45
PTL	40−51 (43)	29−58	47−64	30−47	35−48
PTW	37−55 (45)	38−71	41−65	52−82	45−49
OL	30−52 (39)	26−71	49−71	35−49	30−40
OW	26−54 (37)	39−91	69−88	52−80	37−49
VL	32−64 (51)	57−106	80−97	49−90	44−59
VW	33−63 (45)	44−94	80−94	59−99	37−52
PVS	50−107 (81)	160*	−	111*	50−75
EL	18−23 (21)	17−26	21−24	19−23	21−24
EW	9−16 (12)	10−16	9−13	12−14	9−15
BW/BL (%)	9.2−16.8 (12.6)	11−22	12−16	14*	14−15
SLR (VSL/OSL)	1: 0.92−1.75 (1.41)	1: 1.6*	1: 1.6−1.9	1: 1.5*	1: 1.38−1.6
SWR (VSW/OSW)	1: 0.84−1.66 (1.31)	1: 1.3−1.8	1: 1.5−1.8	1: 1.4−1.6	1: 1.4−1.42
VSW/BWVS (%)	55.1−119 (76)	68*	63−68	60*	64
FO/BL (%)	25.4−35.8 (30.5)	25−34	29−32	31*	31−37
VL/BL (%)	7.9−12.6 (10.6)	11−19	7−12	11*	9
MFL/VSL (%)	42.8−147 (76)	60*	43−76	53*	78
MFW/VSW (%)	20−42.5 (32.6)	18*	20−23	21*	32
MFW/BWVS (%)	12.6−38.7 (25.7)	12*	8−16	13*	20
MFW/MFL (%)	32.2−85.7 (54.7)	33*	31−38	42*	37
LPL/BL (%)	26.7−38.6 (31.7)	30−37	31−34	38*	29−33
LPW/BW (%)	141−287 (170)	143*	113−134	113*	123
LPW/LPL (%)	45.1−74.2 (59.6)	47*	41−63	52*	55

*=Estimated from the published drawing. Mean value in parenthesis. BL body length, NPSG, Number of pseudosegments, BWatVSF Body width at ventral sucker flange, BWatVS Body width at ventral sucker, FO forebody, LPL Last pseudosegment length, LPW Last pseudosegment width, OSL oral sucker length, OSW oral sucker width, VSL ventral sucker length, VSW ventral sucker width, FatVSL Flange at ventral sucker length, FatVSW Flange at ventral sucker width, PHL pharynx length, PHW pharynx width, OEL oesophagus length, SSL Sinus sac length, SSW Sinus sac length width, SVL seminal vesicle length, SVW seminal vesicle width, PPL pars prostatica length, PPW pars prostatica width, ATL anterior testis length, ATW anterior testis width, PTL posterior testis length, PTW posterior testis width, OL ovary length, OW ovary width, VL Vitellarium length, VW Vitellarium width, PVS postvitelline space, EL eggs length, EW eggs width, BW/BL (%) body width as a percentage of body length, Sucker length ratio SLR (vsl/osl) ventral sucker length as a proportion of oral sucker length, Sucker width ratio SWR (vsw/osw) ventral sucker width as a proportion of oral sucker width, VSW/BWVS (%) ventral sucker width as a percentage of body width at the level of the anterior margin of the ventral sucker, FO/BL (%) forebody as a percentage of body length, VL/BL (%) vitellarium length as a percentage of body length, MFL/VSL (%) muscular flange length as a percentage of ventral sucker length, MFW/VSW (%) muscular flange width as a percentage of the width of the ventral sucker, MFW/BWVS (%) muscular flange width as a percentage of body width at the anterior border of the ventral sucker, MFW/MFL (%) muscular flange width as a percentage of length, LPL/BL (%) last pseudosegment length as a percentage of body length, LPW/BW (%) last pseudosegment width as a percentage of body width, LPW/LPL (%) last pseudosegment width as a percentage of its length

Body elongate, gradually tapering anteriorly, with seven pseudosegments. Maximum body width at level of ventral sucker flange, width 9.2−16.8 of body length. Preoral dorsal lip wide (Fig 1d). Two flanges, one located at oral sucker level, and the second at or slightly posterior to ventral sucker, mound-shaped. Oral sucker aperture surrounded by 8 small papillae arranged in four pairs (Fig 1d). Ventral sucker located between the second and third pseudosegment. Forebody 25.4−35.8% of body length. Large single cells in most segments, lateral to body. Pharynx contiguous; prepharynx not observed. Oesophagus thick walled, smaller than pharynx. Caeca with constrictions in pseudosegments, terminating near posterior end, “Drüsenmagen” just posterior to oesophagus.

Testes 2, smooth or irregular, in the last two segments, in tandem, between the caeca. Sinus sac small containing eversible hermaphroditic duct; metraterm short. Seminal vesicle large, elongate, longer than sinus sac, extending to anterior border of ventral sucker. Pars prostatica vesicular, small, difficult to observe, enters sinus-sac as its base. Genital pore median at or near level of anterior septum.

Ovary transversely elongate, pos- testicular, in last major segment, ventral, smooth to irregular, in middle of caeca. Mehlis’ gland conspicuous in some specimens. Vitellarium smooth, 7.9−12.6 of body length, contiguous with ovary. Uterus filling most of partitions, extending posterior to vitellarium but not reaching posterior end. Eggs numerous, small. Excretory vesicle consisting of posterior muscular sac within the last pseudosegment. Pore terminal.

### Remarks

The specimens found in *Mugil curema* from La Carbonera coastal lagoon were identified as *Parasaturnius maurepasi* **n. gen. n. comb.** based on morphology, host and geographical distribution as in the original description (Overstreet, 1977) (Table 2). In addition, following the key to species proposed by Blasco-Costa et al. (2008), our specimens fall in S*. maurepasi* since they possess seven pseudosegments separated by six transverse fibrous septa. However, we found some slight morphological differences with respect to the original description, as the papillae surrounding the oral sucker, since we were able to obtain SEM photomicrographs of the sampled specimens. Moreover, the metrical data suggest specimens found in the present study in Yucatán are overall slightly smaller than the specimens described by Overstreet (1977).

## Molecular data and phylogenetic analysis

**Figure 2.**
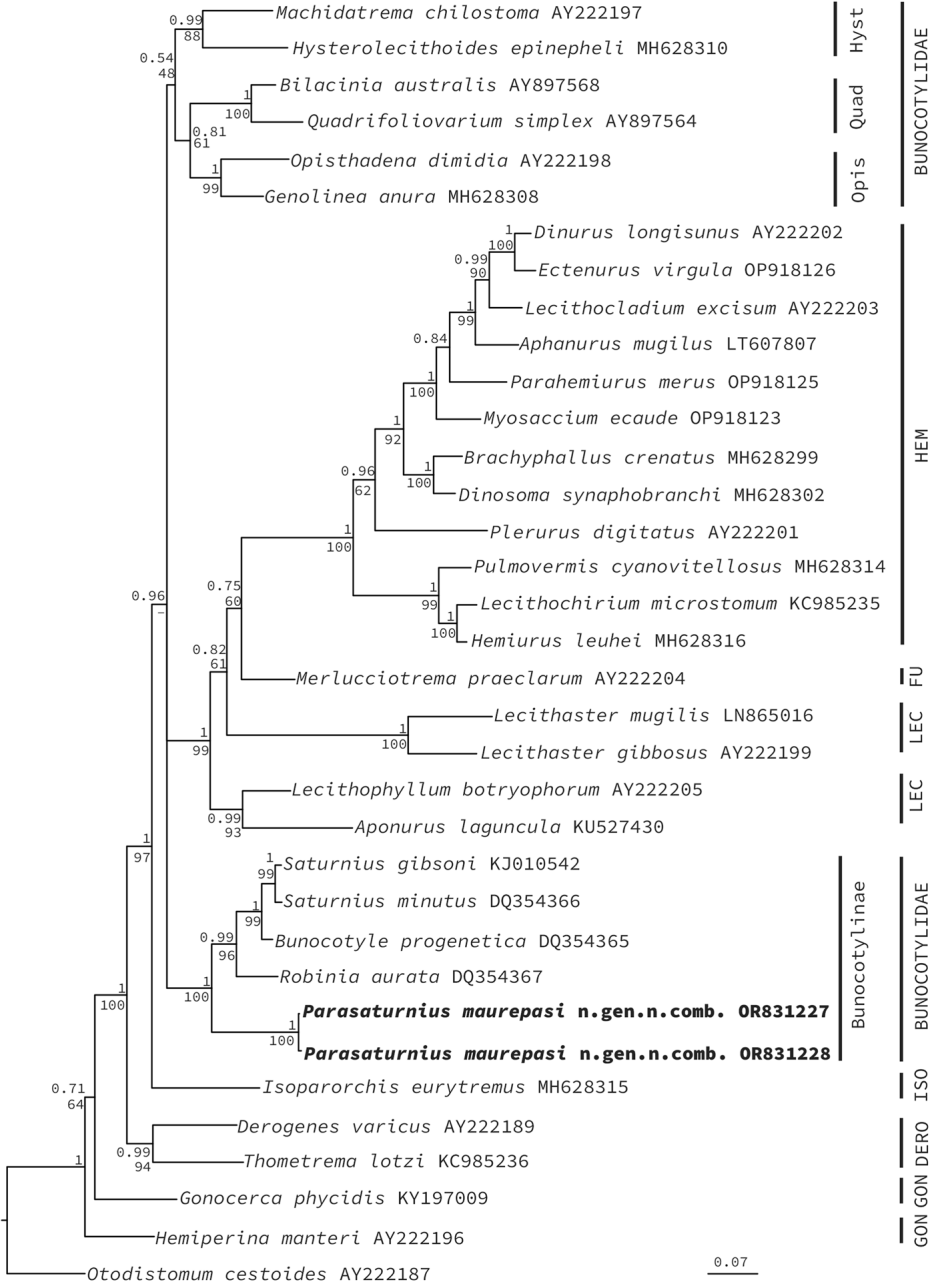
Consensus Bayesian Inference and Maximum Likelihood trees of genera of Hemiuroids inferred with 28S rRNA sequence data; numbers near internal nodes show posterior probabilities (BI) and ML bootstrap clade frequencies. Opis= Opisthadeninae; Quad=Quadrifoliovariinae; Hyst= Hysterolecithinae. HEM= Hemiuridae, ISO=Isoparorchiidae; DERO= Derogenidae; GON= Gonocercidae; LEC= Lecithasteridae; FU=Family Unknown *sensu* Atopkin et al. 2020.

The 28S data set included 35 sequences and comprised 1, 326 nucleotides. The alignment (trimmed to the shortest sequence) included one sequence of Azygiidae used as outgroup. The phylogenetic analyses inferred with ML and BI recovered similar topologies (Fig. 2). The analyses show that the superfamily Hemiuroidea is monophyletic. Within each family, Hemiuridae, Isoparorchiidae, and Derogenidae were recovered as monophyletic, with strong nodal support (1/100; 1/97; 0.99/94). The remaining three families analyzed were not recovered as monophyletic, e. g., Gonocercidae, Lecithasteridae and Bunocotylidae. Bunocotylids were separated in two independent clades, one formed by three subfamilies, Hysterolecithinae, Quadrifoliovariinae and Opisthadeninae, with low support (0.54/48). The second major clade was formed only by Bunocotylinae, with strong support (1/100). Within Bunocotylinae, the two newly generated sequences of *Parasaturnius maurepasi* **n. gen. n. comb.** were nested as the sister group of the three genera contained in the subfamily, e.g., *Robinia, Bunocotyle,* and *Saturnius*, with strong support (1/100) (Fig. 2).

The genetic divergence estimated for 28S for the Bunocotylinae and the other three subfamilies (Hysterolecithinae, Opisthadeninae, Quadrifoliovariinae) ranged from 12.6 to 19.1%. Within Bunocotylinae, the two new isolates of *Parasaturnius maurepasi* **n. gen. n. comb.** varied 10.7-12% with *Saturnius* spp., 10.5-11.5% with *B. progenetica*, and 10.8-11.5 with *R. aurata*. The intraspecific genetic divergence between the two isolates of the new species we described in this study was 0.2% (Table 3).

**Table 3:** Pairwise nucleotide sequence comparisons between taxa of Bunocotylidae for the aligned 28S rDNA sequences (1,326 nt)

Bunocotylidae				Bunocotylinae				
28S	Hysterolecithinae	Opisthadeninae	Quadrifoliovariinae	*Saturnius minutus*	*Saturnius gibsoni*	*Bunocotyle progenetica*	*Robinia aurata*	*Parasaturnius maurepasi* **n. gen. n. comb.**
Hysterolecithinae	12.3	–	–	–	–	–	–	–
Opisthadeninae	13.6–16.5	9.7	–	–	–	–	–	–
Quadrifoliovariinae	12.5–15.2	11.9	6.9	–	–	–	–	–
*Saturnius minutus*	12.6–14.7	13.1–14.8	13.4–15.2	–	–	–	–	–
*Saturnius gibsoni*	12.6–14.7	13.1–14.8	13.4–15.2	1.2	–	–	–	–
*Bunocotyle progenetica*	12.6–14.7	13.1–14.8	13.4–15.2	2.8	2.7	–	–	–
*Robinia aurata*	12.6–14.7	13.1–14.8	13.4–15.2	6.6	6.8	6.6	–	–
*Parasaturnius maurepasi* **n. gen. n. comb.**	16.4–17.4	15.5–19.1	15.5–19.1	10.7–11.3	11.3–12	10.5–11.5	10.8–11.5	**0.2**

In bold is represented the genetic intraspecific divergence

## Discussion

The two newly sequenced specimens of *Parasaturnius maurepasi* **n. gen. n. comb.** were recovered as the sister group of the three previously mentioned genera, with the same topology from previous studies, e.g., *Robinia* as sister of *Saturnius* and *Bunocotyle* (Pankov et al., 2006; Marzoug et al., 2014). Interestingly, even though *Saturnius* and *Parasaturnius* **n. gen.** are morphologically very similar, molecularly they are not closely related and exhibit large genetic divergence of the gene 28S. *Saturnius gibsoni* (KJ010542) and *S. minutus* (DQ354366) are sister taxa to *Bunocotyle progenetica* (DQ354365), with less than 3% of genetic divergence, which is quite low if we consider that divergence between *Robinia aurata* and the three species previously mentioned varies from 6.6 to 6.8% (Table 3). Furthermore, the genetic divergence between *Parasaturnius maurepasi* **n. gen. n. comb.** and the four species was even higher, and ranged from 10.7–12%, which suggest that presence of pseudosegments in *Saturnius* and the new genus are homoplasies in these two genera. Interestingly, these high genetic divergence values between genera are like those reported among other genera of hemiuroid trematodes (Pantoja & Kudlai, 2022).

The original description of *P. maurepasi* **n. gen. n. comb.** was published by Overstreet (1977) (as *Saturnius maurepasi*) from the stomach of *Mugil cephalus* in Ocean Springs, Mississippi, USA. After the original record, the species has been reported across the Atlantic coast of the Americas, as a parasite of *M. liza* in Colombia and Brazil (Romero & Galeano, 1981; Fernandes & Goulart, 1992; Knoff et al., 1997). The species is reported for the first time in Mexico, as a parasite of *Mugil curema* in La Carbonera coastal lagoon, representing new host and new locality records for this parasite. In the same geographic region, a morphologically similar species has been reported, i.e., *S. belizensis.* Fischtal (1977) described *S. belizensis* as a parasite of *M. curema* from Belize, a locality relatively close to La Carbonera, Yucatán, Mexico. It is noteworthy that most probably both, Overstreet (1977) and Fischtal (1977), did not know about each other´s paper describing a new species of *Saturnius* published in the same year. Furthermore, *S. belizensis* was described from only two specimens. In the last taxonomic review of the genus *Saturnius*, Blasco-Costa et al. (2008) studied a paratype of this species and noticed that *S. belizensis* exhibits five pseudogements. Even though our specimens are metrically more similar to *S. belizensis*, the number of pseudosegments in our specimens are seven, similar to that reported by Blasco-Costa et al. (2008) for *S. maurepasi*. Testing the validity of *S. belizensis* will require the generation of 28S DNA sequences from the type locality, and a through morphological study of a larger number of specimens to corroborate the number of pseudosegments. In addition to that, the schematic drawings of *S. maurepasi* from *M. liza* in Brazil shows they possess six pseudosegments; based on host association and geographical distribution, these specimens could indeed represent a separate lineage. Therefore, a detailed morphological study and sequence data are required to corroborate the status of these reports (Fernandes & Goulart, 1992).

Based on the evidence discussed above, *Parasaturnius maurepasi* **n. gen. n. comb.** is here recognized based on molecular data and a detailed morphological analysis. Still, sampling specimens of other valid species is required to be analyzed under a molecular approach, to clarify the species and family status. It is necessary to study further *Saturnius segmentatus,* the type species, which is the only species allocated in the genus *Saturnius* exhibiting morphological traits similar to those of *Robinia*, such as the muscular and conspicuous papillae on the oral sucker. In addition, the cyclocoel is a morphological trait of *Bunocotyle*, however this character is also found in four species of *Saturnius*, i.e*., S. gibsoni, S. minutus, S. dimitrovi* and *S. overstreeti*. In this sense, it would be also necessary to obtain sequence data of *Saturnius* spp. lacking cyclocoel and *Bunocotyle* spp. to test the evolution of morphological traits in Bunocotylinae.

**Comments on the taxonomic status of Bunocotylidae**.

Using molecular data, Sokolov et al. (2018) and Atopkin et al. (2020) resurrected the family Bunocotylidae, and considered it contained the subfamilies Bunocotylinae Opisthadeninae, Hysterolecithinae and Quadrifoliovariinae. However, two recent studies provided evidence in favor of the paraphyly of Bunocotylidae (Louvard et al., 2022; Faltýnkova et al., 2022), although these studies did not include all the genera considered by Atopkin et al. (2020) as members of the family. In our study, after including all the genera of bunocotylids following the concept by Atopkin et al. (2020) we found no conclusive evidence on the interrelationships among members of the families Bunocotylidae, Lecithasteridae and Hemiuridae. The phylogenetic tree yielded a basal polytomy and it might be premature to consider that the genera *Machidatrema* León-Règagnon, 1998, *Hysterolecithoides* Yamaguti, 1934, *Bilacinia* Manter, 1969, *Quadrifoliovarium* Yamaguti, 1965, *Opisthadena* Linton 1910, and *Genolinea* Manter, 1925 (allocated in Hysterolecithinae, Opisthadeninae, Quadrifoliovariinae) belong to Bunocotylidae. Therefore, more sequence data is required to resolve the systematic interrelationships among this hemiuroid group.
